# Comparative efficacy and safety of methoxy polyethylene glycol-epoetin beta and roxadustat in the management of renal anemia in maintenance hemodialysis

**DOI:** 10.3389/fphar.2026.1862251

**Published:** 2026-08-20

**Authors:** Hai-E. Tang, Yan-E. Qiu, Rong-Xiu Ge, Xiao-Jie Lin, Qin Zhao, Qiang Liu

**Affiliations:** Department of Nephrology, The Affiliated Guangdong Second Provincial General Hospital of Jinan University, Guangzhou, Guangdong, China

**Keywords:** CERA, chronic kidney disease, hemodialysis, renal anemia, retrospective study, roxadustat

## Abstract

**Objective:**

This study aimed to evaluate and compare the efficacy and safety of methoxy polyethylene glycol-epoetin beta, a continuous erythropoietin receptor activator (CERA), and roxadustat in the management of renal anemia among patients undergoing maintenance hemodialysis (MHD).

**Methods:**

A retrospective analysis was conducted involving 60 patients with renal anemia receiving MHD at a single center between December 2024 and June 2025. Participants were assigned to one of two treatment groups (n = 30 per group). The CERA group received intravenous methoxy polyethylene glycol-epoetin beta at a starting dose of 100 μg, administered every 2–6 weeks with dose adjustments based on hemoglobin (Hb) levels. The roxadustat group received oral roxadustat capsules, initiated at 100 mg three times weekly, with dose modifications ranging from 20 to 150 mg according to Hb responses. After 6 months of treatment, the primary outcome was the between-group difference in Hb change from baseline to month 6. Secondary outcomes included Hb target attainment, defined strictly as 110–130 g/L, and hypertension rate. Missing baseline covariates were imputed using k-nearest neighbors (k = 10) before overlap weighting. The overlap-weighting model included baseline Hb, age, sex, primary kidney disease, hypertension at baseline, baseline parathyroid hormone, body weight, baseline C-reactive protein, dialysis vintage, prior ESA type/dose, baseline ferritin, and baseline transferrin. The weighted Hb outcome model was a mixed model for repeated measures including treatment group, time, treatment-by-time interaction, and baseline Hb. Weighted generalized estimating equation models were used for binary repeated outcomes.

**Results:**

After overlap weighting, baseline Hb was 93.0 (14.49) g/L in the roxadustat group and 93.0 (15.79) g/L in the CERA group. At month 6, weighted mean Hb was 103.4 (14.87) g/L and 111.7 (10.23) g/L, respectively. The 6-month difference in least-squares mean Hb change for CERA versus roxadustat was 8.27 (1.87, 14.67) (P = 0.012). Weighted Hb target attainment at month 6 was 37.9% in the roxadustat group and 50.6% in the CERA group; the month 6 treatment-by-time interaction RR was 1.16 (0.26–5.11) (P = 0.846). Weighted hypertension rates at month 6 were 60.7% and 57.5%, respectively; the month 6 treatment-by-time interaction RR was 0.95 (0.57–1.57) (P = 0.834).

**Conclusion:**

In this small retrospective cohort, CERA was associated with a larger 6-month Hb increase than roxadustat after overlap weighting. Hb target attainment and hypertension-rate comparisons did not show statistically significant treatment-by-time interaction effects. Because treatment allocation reflected clinical practice and patient preference, residual selection bias remains an important competing explanation.

## 1 Introduction

Anemia is a central and clinically consequential complication of chronic kidney disease (CKD), particularly among patients receiving maintenance hemodialysis. Contemporary guidelines emphasize individualized hemoglobin (Hb) monitoring, iron assessment, avoidance of excessive Hb correction, and judicious use of erythropoiesis-stimulating agents (ESAs) and hypoxia-inducible factor prolyl hydroxylase inhibitors (HIF-PHIs) (Kidney Disease: Improving Global Outcomes KDIGO Anemia Work Group et al., 2026; Babitt et al., 2026). The biological basis of CKD anemia is multifactorial, involving impaired erythropoietin production, shortened red-cell survival, and iron-restricted erythropoiesis (Babitt and Lin, 2012). Population data and clinical reviews document the burden of anemia across CKD stages and in dialysis care (Stauffer and Fan, 2014; Fishbane and Spinowitz, 2018). Inflammation and hepcidin-mediated changes in iron availability further contribute to renal-anemia biology (Babitt and Lin, 2012; Weiss and Goodnough, 2005). In hemodialysis populations, iron therapy and ESA dosing are therefore inseparable parts of anemia management, and intravenous iron strategies can influence ESA requirements and Hb control (Macdougall et al., 2019).

The historical ESA literature also defines an important safety boundary for interpreting anemia-treatment studies. Trials targeting higher hematocrit or near-normal Hb levels in CKD or dialysis populations raised concerns about cardiovascular and thrombotic risk and did not establish broad benefit from full normalization of Hb (Besarab et al., 1998; Singh et al., 2006; Pfeffer et al., 2009). These findings are directly relevant to the present analysis because Hb target attainment was defined as the prespecified range of 110–130 g/L, rather than simply Hb of at least 110 g/L. Patients with Hb values above 130 g/L were therefore not classified as target achievers.

HIF-PHIs have expanded therapeutic options for renal anemia by stimulating endogenous erythropoietin pathways and modifying iron metabolism (Ku et al., 2023; Sanghani and Haase, 2019). Roxadustat has shown efficacy in dialysis-dependent CKD trials, including a Chinese phase 3 dialysis trial and the ROCKIES trial (Chen et al., 2019b; Fishbane et al., 2022). These data support roxadustat as an effective anemia therapy in trial settings, but they do not eliminate the need to understand real-world outcomes when treatment allocation is influenced by patient preference, prior ESA exposure, inflammation, iron status, dialysis vintage, and other clinical factors.

Methoxy polyethylene glycol-epoetin beta, also known as continuous erythropoietin receptor activator (CERA) and marketed as Mircera, is a long-acting ESA designed for extended dosing intervals. Randomized dialysis studies have shown that CERA can maintain Hb control with extended dosing schedules (Klinger et al., 2007; Carrera et al., 2010). In real-world dialysis care, however, physician and patient choices between oral roxadustat and injectable ESA therapy can be influenced by prior treatment, anticipated responsiveness, inflammation, iron status, and dialysis-unit practice.

## 2 Materials and methods

### 2.1 Study participants

This retrospective study included patients who underwent maintenance hemodialysis (MHD) at our center between December 2024 and June 2025 and had received treatment with either CERA or roxadustat for at least 6 months. Baseline Hb was defined as the value obtained before treatment initiation, with the treatment initiation date serving as the baseline. Although patient enrollment occurred between December 2024 and June 2025, the study retrospectively analyzed data from the treatment baseline onward.

#### 2.1.1 Inclusion criteria

(1) Patients diagnosed with CKD undergoing MHD and presenting with renal anemia; (2) a dialysis duration of at least 1 week; (3) age greater than 18 years; (4) C.E.R.A. and roxadustat were administered for at least 6 months.

#### 2.1.2 Exclusion criteria

Patients were excluded if red blood cell transfusion had been received within the 8 weeks prior to enrollment; if active malignancy was present; or if there were uncontrolled or chronic immunologic disorders, severe infections, hemolytic anemia, pure red cell aplasia, severe hepatic or cardiac dysfunction. Pregnant or lactating women, as well as women of childbearing potential not using contraception, were excluded.

#### 2.1.3 Study design and population

This retrospective comparative cohort study included 60 maintenance hemodialysis patients with renal anemia from the final analysis dataset and was reported in accordance with the STROBE framework for observational studies (von Elm et al., 2007). Patients were grouped according to the anemia treatment received in routine care: roxadustat (30 patients) or methoxy polyethylene glycol-epoetin beta (Mircera; 30 patients). The analysis used baseline, month 3, and month 6 measurements. For the overlap-weighted primary analysis, patients were required to have a treatment-group assignment and Hb measurements at baseline, month 3, and month 6; 60 patients were included after data preparation.

Baseline variables extracted for description included age, sex, primary kidney disease, baseline Hb, Hb target status, hypertension at baseline, Kt/V, baseline PTH, body weight, baseline CRP, baseline ferritin, baseline TRF, baseline albumin, dialysis vintage, prior ESA type/dose, and ACEI/ARB dose. Prior ESA information was handled as a single type/dose variable. Its levels were labeled to identify ESA-naive patients and ESA-switching patients within the same baseline category.

### 2.2 Sample size calculation

This is a retrospective clinical study with the change in Hb level after treatment designated as the primary endpoint. Parameter settings were derived from a multicenter, randomized, open-label phase III head-to-head non-inferiority trial conducted in China that compared roxadustat with epoetin alfa (standard erythropoiesis-stimulating agent). The expected mean change in Hb was 5 g/L in the epoetin alfa group and 7 g/L in the roxadustat group, yielding a mean intergroup difference (Δ) of 2 g/L with a pooled standard deviation of 1.06. A conservative pooled standard deviation of 1.10 g/dL was adopted to avoid underestimation of data variability. A one-sided significance level of α = 0.05 and statistical power of 90% were specified. Based on the sample size formula for two independent samples t-test, the theoretical sample size without accounting for loss to follow-up was calculated to be 6 subjects per group. Review of the clinical database revealed 30 eligible cases in the epoetin alfa group and another 30 eligible cases in the roxadustat group, which was sufficient to meet the required statistical power. All sample size calculations were validated using PASS 2025 software, and the outputs were consistent with manual computation.

### 2.3 Study methods

#### 2.3.1 Treatment protocols

##### 2.3.1.1 CERA dosing and iron supplementation

In the CERA group, methoxy polyethylene glycol-epoetin beta injection (Mircera, Alebund Pharmaceuticals Co., Ltd.; approval number H1142000174; 100 μg/0.3 mL per vial) was administered. A uniform initial intravenous dose of 100 μg was selected due to limited clinical experience with this formulation in China. Since only the 100 μg specification of CERA (Mircera) is available in our hospital, we initiated treatment at a low starting dose of 100 μg every 4 weeks as recommended in the drug instructions. Based on serial hemoglobin monitoring results, we dynamically adjusted the administration interval to address the limitation of the fixed single dose strength.

Hb levels were monitored 4 weeks after initiation during the initial treatment phase. If the Hb response was deemed inadequate, defined as an increase of less than 10 g/L within 4 weeks or a decline below baseline to <90 g/L, the dosing interval was shortened to 2 weeks. For participants with baseline Hb levels between 90 and 100 g/L, the dosing interval was shortened to 3 weeks, continuing until an increase of ≥10 g/L from baseline was achieved and Hb levels were maintained between 110 and 130 g/L. In cases where Hb exceeded 130 g/L or increased by more than 20 g/L within 4 weeks, the dosing interval was extended to 6 weeks without interruption. If Hb levels rose above 140 g/L, treatment was temporarily discontinued until levels fell below 130 g/L, after which therapy was resumed at an interval extended by 2 weeks compared with the previous cycle.

Once Hb stabilization was achieved, monitoring was conducted every 1–2 months, and treatment was continued for a total duration of 6 months. In accordance with the Japanese Society for Dialysis Therapy guidelines for renal anemia management (Yamam et al., 2015), iron supplementation was initiated when serum ferritin was <100 ng/mL and transferrin saturation (TSAT) was <20%.

Iron supplementation was primarily administered orally using iron polysaccharide complex capsules (SPH Qingdao Growful Pharmaceutical Co., Ltd.; approval number H1142000155; 150 mg per capsule). Dosage and administration: If the Hb level is 100–110 g/L, the dose is 150 mg once daily; if Hb ≤ 100 g/L, the dose is 300 mg once daily. The medication should be swallowed whole with warm water after meals. Iron status shall be reassessed after 1–3 months. If target iron levels are not achieved or oral iron is not tolerated, intravenous iron should be initiated. In cases of severe iron deficiency, intravenous iron supplementation should be administered directly. Intravenous iron sucrose was administered at 40 mg once weekly for 5 weeks, followed by reassessment.

##### 2.3.1.2 Roxadustat dosing and iron supplementation

Roxadustat capsules (Evrenzo, AstraZeneca Pharmaceuticals (China) Co., Ltd.; approval number H1142000113; 50 mg or 20 mg per capsule) were administered in the roxadustat group. Based on clinical experience and safety data obtained at the participating hemodialysis center, a standardized initial oral dose of 100 mg was prescribed three times per week, taken either before or after meals. Available dose strengths included 20, 40, 50, 70, 100, 120, and 150 mg.

Hb levels were monitored every 1–3 months. Dose adjustments were made stepwise in response to Hb levels. In cases of inadequate Hb response, defined as an increase of less than 10 g/L within 4 weeks or a decrease to <90 g/L, dose escalation was implemented stepwise until the target Hb range was achieved. If Hb exceeded 130 g/L or increased by more than 20 g/L within 4 weeks, stepwise dose reduction was undertaken. When Hb levels rose above 140 g/L, treatment was temporarily discontinued and Hb was reassessed every 2 weeks. Once Hb decreased to below 130 g/L, treatment was resumed at one dose tier lower than the previous level. Therapy was maintained for a total of 6 months.

Iron supplementation protocols were identical to those applied in the CERA group.

##### 2.3.1.3 Blood sampling, evaluation, and subgroup analysis

To support anemia management and assess iron status, complete blood counts including white blood cells, red blood cells, Hb, and hematocrit were conducted at intervals of 2–4 weeks throughout the 6-month treatment period. Nutritional markers, including serum albumin, and inflammatory indicators, such as C-reactive protein (CRP), were measured every 3 months. Parameters related to iron metabolism including serum iron, unsaturated iron-binding capacity, serum ferritin, and TSAT were assessed every 6 months.

#### 2.3.2 Outcome measures

The primary endpoint was the between-group difference in Hb change from baseline to month 6. Hb values at baseline, month 3, and month 6 were also summarized descriptively before and after overlap weighting. Hb target attainment was defined as Hb from 110 to 130 g/L, inclusive. Values below 110 g/L and values above 130 g/L were both classified as not attaining the target range.

The target blood pressure for hemodialysis patients should be individualized, taking into account age, blood pressure profiles before and after dialysis, blood volume, comorbidities and other factors (Consensus Expert Panel on the Management of Hypertension in Chronic Kidney Disease, 2023). For patients aged under 60 years, the target range is 90 ≤ SBP <140 mmHg and 60 ≤ DBP <90 mmHg; for patients aged 60 years or older, the target range is 90 ≤ SBP <160 mmHg and 60 ≤ DBP <90 mmHg. Since the subjects of this study are hemodialysis patients, Hypertension was defined using age-specific hemodialysis thresholds. For patients older than 60 years, systolic blood pressure of at least 160 mm Hg or diastolic blood pressure of at least 100 mmHg was considered hypertension. For patients 60 years or younger, systolic blood pressure of at least 140 mm Hg or diastolic blood pressure of at least 90 mmHg was considered hypertension. Meeting either the systolic or the diastolic threshold was sufficient for classification as hypertension.

Roxadustat adherence was evaluated among roxadustat-treated patients. Patient-level adherence was calculated as cumulative dispensed dose divided by cumulative prescribed dose. Dispensed dose was reconstructed from tablet strength and tablet counts, and prescribed dose was reconstructed from the recorded per-dose prescription, recommended dose, and inferred administration count. Dose adjustment burden was summarized by median, quartiles, range, and frequencies for 0, 1, 2, 3, and 4 adjustments. Recorded complications were summarized descriptively because event counts were sparse.

#### 2.3.3 Statistical analysis

Continuous variables were summarized as mean (SD), weighted mean (weighted SD), or median (Q1, Q3), as appropriate. Categorical variables were summarized as counts and percentages before weighting and as weighted percentages after overlap weighting. For descriptive binary outcomes, unweighted rates retained the observed event count and denominator, whereas overlap-weighted rates were reported as weighted percentages without n/N. Weighted 95% CIs for binary descriptive estimates used the effective sample size implied by the overlap weights.

Missing baseline covariates used in the weighting model were imputed by k-nearest neighbors with k = 10. The imputation was applied to baseline covariates used for propensity-score estimation; outcome variables were not imputed. Overlap weights targeting the average treatment effect in the overlap population (ATO) were estimated using an unstabilized logistic propensity-score model with a logit link and no weight trimming. Propensity-score methods were used to reduce measured confounding and to assess covariate balance (Austin, 2011; Austin, 2009). Overlap weighting was selected because it emphasizes the covariate-overlap population and reduces the influence of patients with extreme propensity scores (Li et al., 2019; Thomas et al., 2020). The propensity-score model included baseline Hb, age, sex, primary kidney disease, hypertension at baseline, baseline PTH, body weight, baseline CRP, dialysis vintage, prior ESA type/dose, baseline ferritin, and baseline TRF. Baseline transferrin saturation was not available in the dataset; therefore, TRF was used as the transferrin-related baseline variable available for analysis.

Baseline balance was evaluated with standardized mean differences (SMDs). For categorical variables, the global SMD was defined as the maximum absolute SMD across category levels. All variables displayed in the baseline table were described before and after weighting; variables used in the propensity-score model were identified in the table footnote.

The weighted primary Hb analysis used a mixed model for repeated measures (MMRM) for Hb change from baseline at months 3 and 6. The model included treatment group, time, treatment-by-time interaction, baseline Hb, and an unstructured within-patient covariance matrix for visit. Overlap weights were applied in the outcome model, and Kenward-Roger degrees-of-freedom adjustment was used. Other covariates balanced through overlap weighting were not additionally adjusted in the weighted outcome model.

Binary repeated outcomes were analyzed using overlap-weighted generalized estimating equation (GEE) models with a Poisson distribution, log link, treatment group, time, treatment-by-time interaction, and an exchangeable working correlation structure; this modeling choice was used to report risk ratios rather than odds ratios for binary outcomes (Zou, 2004). Risk ratios (RRs), 95% CIs, and P values are reported. The primary binary contrast was the treatment-by-time interaction at month 6.

Unweighted models were retained as sensitivity analyses and are presented in the Supplementary Material. The unweighted multivariable Hb MMRM included treatment group, time, treatment-by-time interaction, baseline Hb, age, sex, baseline PTH, baseline CRP, dialysis vintage, prior ESA type/dose, baseline ferritin, and baseline TRF. The unweighted binary GEE sensitivity models included treatment group, time, and treatment-by-time interaction without additional covariates.

## 3 Results

### 3.1 Baseline characteristics

The final analysis included 60 patients: 30 in the roxadustat group and 30 in the CERA group. Before imputation, 7 patients had at least one missing baseline covariate used in the weighting model. After k-nearest-neighbor imputation with k = 10, all 60 patients were retained for overlap weighting and repeated-measures analyses.

Baseline covariates before and after overlap weighting are shown in Table 1. The prior ESA type/dose field also identifies ESA-naive and ESA-switching status. In the roxadustat group, 11 (36.7%) were ESA-naive and 19 (63.3%) were ESA-switching; in the Mircera group, 8 (26.7%) were ESA-naive and 22 (73.3%) were ESA-switching.

**TABLE 1:** Baseline covariates before and after overlap weighting (ATO).

Index	Unweighted			Weighted^a^		
	Roxadustat N = 30	Mircera N = 30	SMD^b^	Roxadustat	Mircera	SMD^b^
Age, years, mean ± SD^c^	57 ± 10	60 ± 16	0.198	58 ± 11	58 ± 16	0.000
Female, n (%)^c^	6 (20%)	11 (37%)	0.167	32%	32%	0.000
Primary kidney disease, n (%)^c^	​	​	0.167	​	​	0.000
Diabetic nephropathy	12 (40%)	7 (23%)	​	38%	38%	​
Glomerulonephritis	8 (27%)	11 (37%)	​	27%	27%	​
Hypertensive nephropathy	4 (13%)	3 (10%)	​	11%	11%	​
Polycystic kidney disease	3 (10%)	3 (10%)	​	9.6%	9.6%	​
Other	3 (10%)	6 (20%)	​	14%	14%	​
Baseline Hb, g/L, mean ± SD^c^	96 ± 15	97 ± 16	0.033	93 ± 15	93 ± 16	0.000
Baseline Hb target (110–130 g/L), n (%)	7 (23%)	6 (20%)	0.033	14%	16%	0.021
Hypertension at baseline, n (%)^c^	21 (70%)	16 (53%)	0.167	61%	61%	0.000
Kt/V, mean ± SD	1.40 ± 0.19	1.44 ± 0.37	0.117	1.44 ± 0.21	1.35 ± 0.34	−0.317
Baseline PTH, pg/mL, mean ± SD^c^	271 ± 213	535 ± 512	1.01	313 ± 227	313 ± 296	0.000
Body weight, kg, mean ± SD^c^	66 ± 11	60 ± 15	−0.437	64 ± 12	64 ± 15	0.000
Baseline CRP, mg/L, mean ± SD^c^	20 ± 66	10 ± 16	−0.417	9 ± 32	9 ± 15	0.000
Baseline ferritin, ng/mL, mean ± SD^c^	234 ± 222	184 ± 153	−0.360	183 ± 150	183 ± 130	0.000
Baseline TRF, g/L, mean ± SD^c^	1.83 ± 0.29	1.81 ± 0.26	−0.045	1.82 ± 0.31	1.82 ± 0.27	0.000
Baseline albumin, g/L, mean ± SD	36.3 ± 4.0	35.5 ± 5.0	−0.161	36.1 ± 4.4	35.1 ± 5.3	−0.215
Dialysis vintage, months, mean ± SD^c^	24 ± 20	28 ± 31	0.165	21 ± 18	21 ± 23	0.000
Prior ESA type/dose, n (%)^c^	​	​	0.100	​	​	0.000
No prior ESA (ESA-naive)	11 (37%)	8 (27%)	​	39%	39%	​
rhEPO 10,000 IU/week (ESA-switching)	13 (43%)	15 (50%)	​	44%	44%	​
Darbepoetin alfa 40 ug/week (ESA-switching)	4 (13%)	6 (20%)	​	14%	14%	​
Other (ESA-switching)	2 (6.7%)	1 (3.3%)	​	3.7%	3.7%	​
ACEI/ARB dose, mg/day, mean ± SD	160 ± 107	107 ± 101	−0.500	169 ± 103	130 ± 108	−0.364

Statistics are Mean ± SD or n (%) unless otherwise indicated.

^a^ After overlap weighting, weighted values are reported as weighted percentages or weighted summary statistics; raw counts are not reported for the weighted sample.

^b^ SMD, standardized mean difference. For continuous variables, SMD was calculated as the between-group difference in means divided by the pooled SD. For categorical variables, the global SMD was defined as the maximum absolute SMD across category levels.

^c^ Variables included in the propensity score model used to estimate overlap weights.

The sums of overlap weights were 11.075 in the roxadustat group and 11.075 in the Mircera group. The corresponding effective sample sizes were 22.962 and 21.152, respectively, and the observed weight range was 0.0145–0.8487. Among variables included in the propensity-score model, the maximum absolute SMD after weighting was 0.000. Across all variables displayed in Table 1, the maximum post-weighting absolute SMD was 0.364; 3 displayed variables had post-weighting absolute SMDs greater than 0.1.

### 3.2 Roxadustat dose adherence

Among roxadustat-treated patients, patient-level dose information was available for 30/30 patients, and 89/90 time points were analyzable. The median (Q1, Q3) cumulative prescribed dose was 3,800 (3,800, 4,000) mg per patient, and the median cumulative dispensed dose was 3,800 (3,732, 4,000) mg per patient (Table 2). The mean (SD) patient-level dispensed/prescribed dose ratio was 98.3% (7.1%), while the median (Q1, Q3) ratio was 100.0% (100.0%, 100.0%); the observed range was 67.5%–109.2%.

**TABLE 2:** Roxadustat dose adherence and dose adjustments.

Item	Roxadustat (N = 30)
Analyzable patients, n/N	30/30
Analyzable dose records, n/N	89/90 time points
Cumulative prescribed dose, mg/patient, median (Q1, Q3)	3,800 (3,800, 4,000)
Cumulative dispensed dose, mg/patient, median (Q1, Q3)	3,800 (3,732, 4,000)
Dispensed/prescribed dose ratio, mean (SD)	98.3% (7.1%)
Dispensed/prescribed dose ratio, median (Q1, Q3)	100.0% (100.0%, 100.0%)
Dispensed/prescribed dose ratio, range	67.5%–109.2%
Ratio ≥ 80%, n (%)	29 (96.7%)
Ratio ≥ 90%, n (%)	27 (90.0%)
Number of dose adjustments, median (Q1, Q3)	0 (0, 1)
Dose adjustments: 0 times, n (%)	18 (60.0%)
Dose adjustments: 1 time, n (%)	7 (23.3%)
Dose adjustments: 2 times, n (%)	3 (10.0%)
Dose adjustments: 3 times, n (%)	1 (3.3%)
Dose adjustments: 4 times, n (%)	1 (3.3%)
Dose adjustments, range	0–4

Adherence was summarized at the patient level as cumulative dispensed dose divided by cumulative prescribed dose. Dispensed dose was derived from tablet strength and tablet counts; prescribed dose was derived from recorded per-dose prescriptions, recommended doses, and inferred administration counts.

At the patient level, 29 (96.7%) had a dispensed/prescribed dose ratio of at least 80%, and 27 (90.0%) had a ratio of at least 90%. The median (Q1, Q3) number of dose adjustments was 0 (0, 1), with a range of 0–4. Dose adjustment counts were 0 in 18 patients, 1 in 7 patients, 2 in 3 patients, 3 in 1 patient, and 4 in 1 patient.

### 3.3 Efficacy analysis of Hb levels at 6 months

**FIGURE 1:**
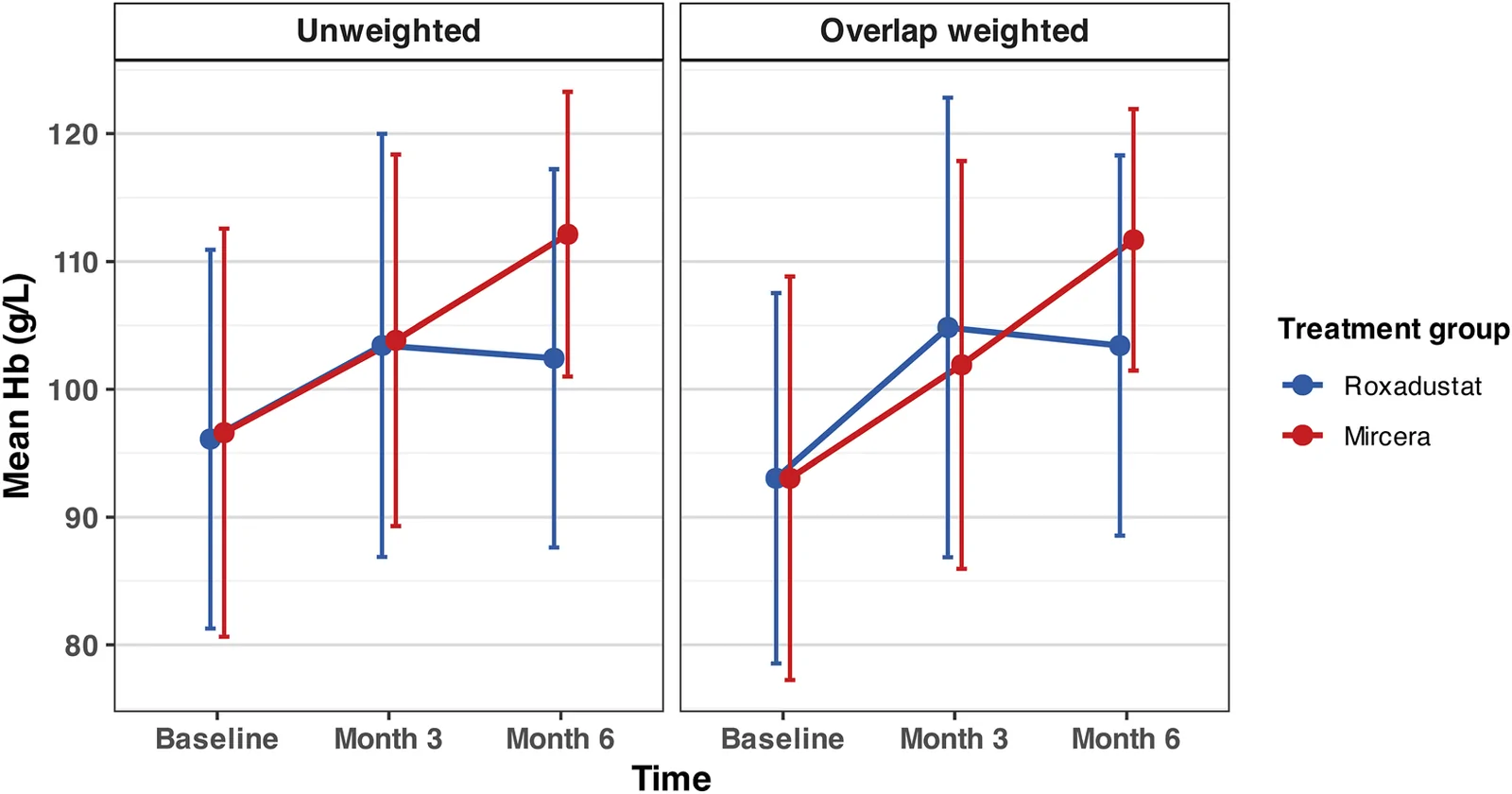
Mean hemoglobin levels over the 6-month treatment period in both groups.

Hb levels at each time point are shown in Table 3 and Figure 1. In the unweighted data, baseline Hb was 96.1 (14.82) g/L in the roxadustat group and 96.6 (15.97) g/L in the Mircera group. At month 6, the corresponding unweighted Hb means were 102.4 (14.80) g/L and 112.1 (11.14) g/L.

**TABLE 3:** Hb level at each time point before and after overlap weighting.

Treatment group	Time point	n	Unweighted mean (SD)	Overlap-weighted mean (weighted SD)
Roxadustat	Baseline	30	96.1 (14.82)	93.0 (14.49)
Roxadustat	Month 3	30	103.4 (16.55)	104.8 (17.97)
Roxadustat	Month 6	30	102.4 (14.80)	103.4 (14.87)
Mircera	Baseline	30	96.6 (15.97)	93.0 (15.79)
Mircera	Month 3	30	103.8 (14.53)	101.9 (15.96)
Mircera	Month 6	30	112.1 (11.14)	111.7 (10.23)

n indicates the unweighted number of available observations. Weighted means and weighted SDs were estimated using ATO overlap weights.

After overlap weighting, baseline Hb was 93.0 (14.49) g/L in the roxadustat group and 93.0 (15.79) g/L in the Mircera group. Weighted mean Hb at month 3 was 104.8 (17.97) g/L in the roxadustat group and 101.9 (15.96) g/L in the Mircera group. At month 6, weighted mean Hb was 103.4 (14.87) g/L in the roxadustat group and 111.7 (10.23) g/L in the Mircera group.

In the overlap-weighted MMRM, the weighted observed mean change from baseline to month 6 was 10.4 (weighted SD 16.54) g/L in the roxadustat group and 18.7 (weighted SD 16.20) g/L in the Mircera group (Table 4). The model-based LS mean change was 8.40 (SE 2.283) g/L for roxadustat and 16.67 (SE 2.283) g/L for Mircera. The 6-month LS mean difference in Hb change for Mircera versus roxadustat was 8.27 (1.87, 14.67) (P = 0.012).

**TABLE 4:** Overlap-weighted efficacy analysis for Hb change at 6 months.

Characteristic	Roxadustat (N = 30)	Mircera (N = 30)
Baseline Hb (g/L)		
n	30	30
Weighted mean (weighted SD)	93.0 (14.49)	93.0 (15.79)
6-month Hb (g/L)		
n	30	30
Weighted mean (weighted SD)	103.4 (14.87)	111.7 (10.23)
Change from baseline		
n	30	30
Weighted mean (weighted SD)	10.4 (16.54)	18.7 (16.20)
LS mean change (SE), ^a^	8.40 (2.283)	16.67 (2.283)
Between-group comparison		
Mircera - roxadustat	​	​
Difference in LS mean (95% CI)^a^	8.27 (1.87, 14.67)	​
P Value	0.012	​

^a^ The analysis was based on an overlap-weighted mixed model for repeated measures including Hb changes at months 3 and 6; this table displays the prespecified 6-month primary endpoint. The outcome model included treatment group, time, treatment-by-time interaction, and baseline Hb. Covariates balanced through overlap weighting were not additionally adjusted. CI, confidence interval; Hb = hemoglobin; LS, least squares; SD, standard deviation.

### 3.4 Proportion of patients achieving Hb (110–130 g/L) after 6 months

**FIGURE 2:**
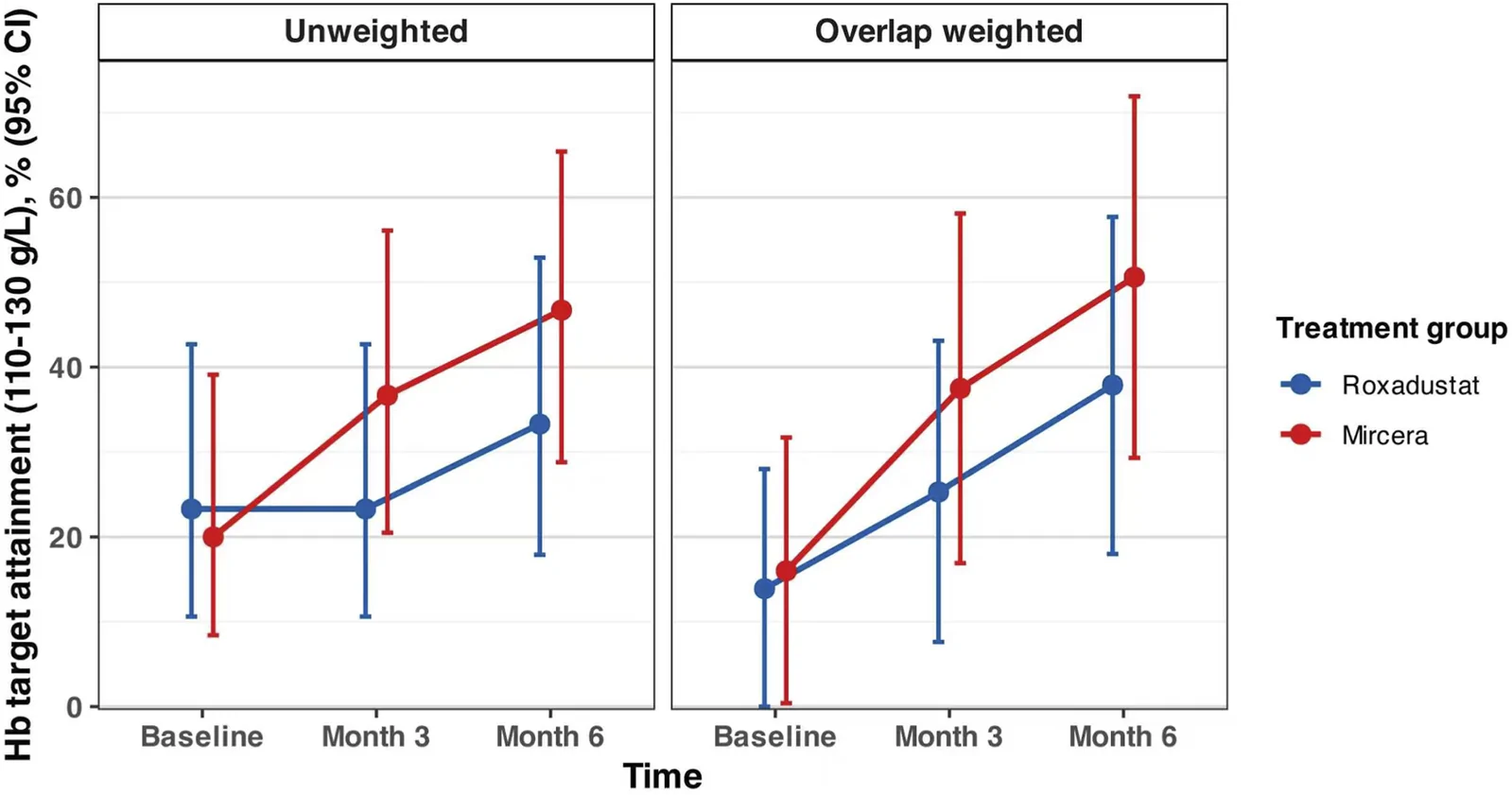
Hemoglobin target attainment rate over time in both groups. Error bars representing the 95% confidence intervals for each time point are shown in Figure 2.

Hb target attainment, defined as 110–130 g/L, is summarized in Table 5 and Figure 2. In the unweighted data, target attainment was 7/30 (23.3%) in the roxadustat group and 6/30 (20.0%) in the Mircera group at baseline. At month 6, target attainment was 10/30 (33.3%) and 14/30 (46.7%), respectively.

**TABLE 5:** Hb target attainment (110–130 g/L) before and after overlap weighting.

Treatment group	Time point	Unweighted rate (n/N)	Unweighted 95% CI	Overlap-weighted rate	Weighted 95% CI
Roxadustat	Baseline	23.3% (7/30)	10.6–42.7	13.9%	0.0–28.0
Roxadustat	Month 3	23.3% (7/30)	10.6–42.7	25.3%	7.6–43.1
Roxadustat	Month 6	33.3% (10/30)	17.9–52.9	37.9%	18.0–57.7
Mircera	Baseline	20.0% (6/30)	8.4–39.1	16.0%	0.4–31.7
Mircera	Month 3	36.7% (11/30)	20.5–56.1	37.5%	16.9–58.1
Mircera	Month 6	46.7% (14/30)	28.8–65.4	50.6%	29.3–71.9

Unweighted rates are observed proportions and retain the original event count/available denominator. Overlap-weighted rates are ATO-weighted estimates and are presented without n/N. CI, confidence interval.

After overlap weighting, target attainment was 13.9% in the roxadustat group and 16.0% in the Mircera group at baseline. At month 3, the weighted rates were 25.3% and 37.5%, respectively; at month 6, they were 37.9% and 50.6%. In the weighted GEE model, the month 6 time effect in the roxadustat reference group had an RR of 2.72 (0.98–7.57) (P = 0.055). The month 3 treatment-by-time interaction RR was 1.28 (0.27–6.06) (P = 0.753), and the prespecified month 6 treatment-by-time interaction RR was 1.16 (0.26–5.11) (P = 0.846) (Table 6).

**TABLE 6:** Overlap-weighted GEE model for Hb target attainment (110–130 g/L).

Effect	Weighted RR (95% CI)^a^	P Value
Group effect (Mircera vs. roxadustat)	1.15 (0.34–3.96)	0.821
Time effect (month 3 vs. baseline)	1.82 (0.59–5.62)	0.295
Time effect (month 6 vs. baseline)	2.72 (0.98–7.57)	0.055
Group x time (Mircera vs. roxadustat, month 3 vs. baseline)	1.28 (0.27–6.06)	0.753
Group x time (Mircera vs. roxadustat, month 6 vs. baseline)	1.16 (0.26–5.11)	0.846

^a^ The model was an overlap-weighted GEE, model including treatment group, time, and treatment-by-time interaction; covariates balanced through overlap weighting were not additionally adjusted.

The group effect represents the between-group difference at baseline; time effects represent changes from baseline in the reference group; group-by-time terms represent difference-in-differences. The month 6 interaction was the prespecified primary contrast. CI, confidence interval; GEE, generalized estimating equations; RR, risk ratio; exchangeable correlation structure.

### 3.5 Safety

**FIGURE 3:**
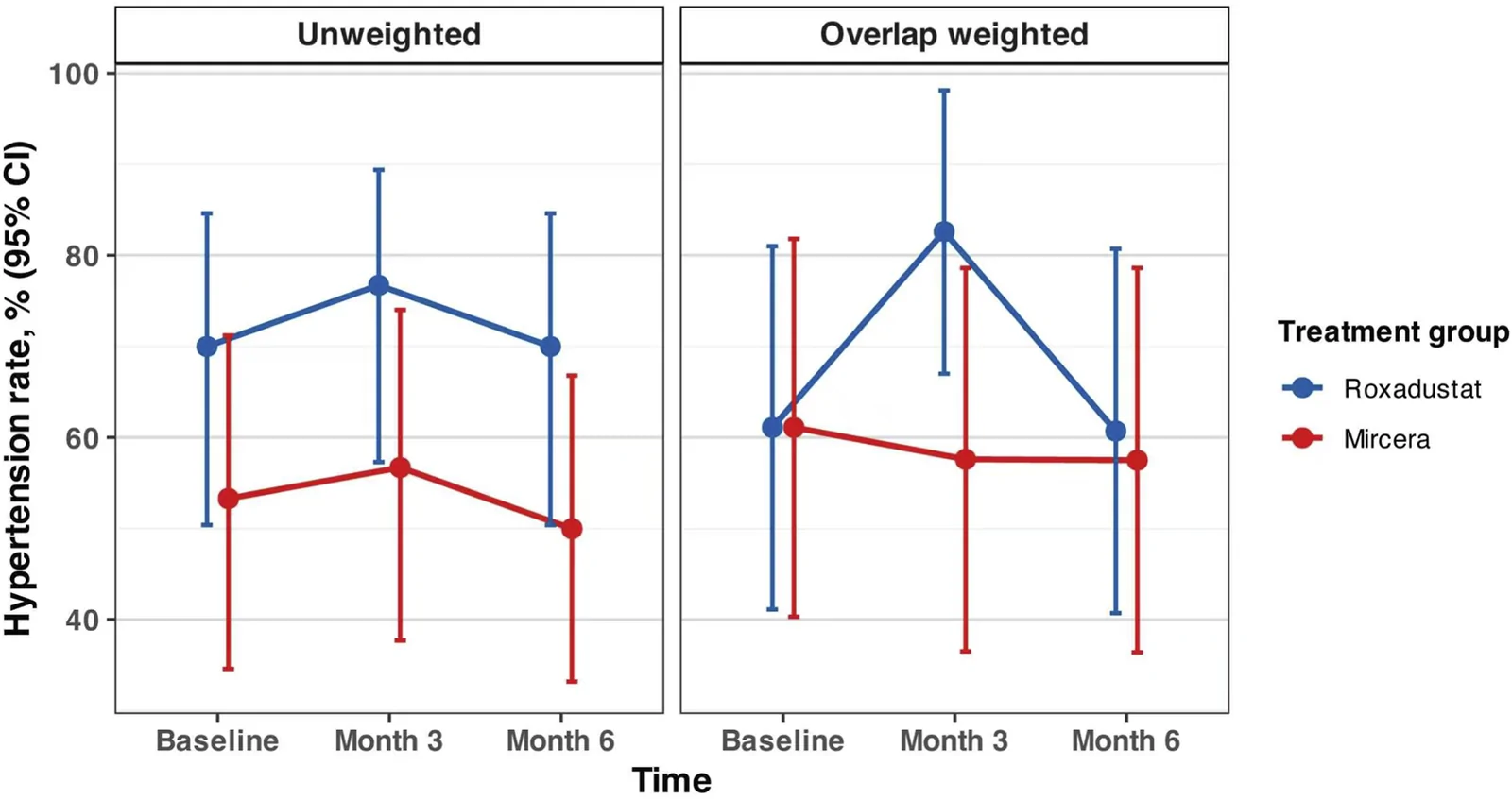
Hypertension incidence over time in both groups.

Hypertension rates are summarized in Table 7 and Figure 3. In the unweighted data, hypertension was present in 21/30 (70.0%) roxadustat-treated patients and 16/30 (53.3%) Mircera-treated patients at baseline. At month 6, hypertension was present in 21/30 (70.0%) and 15/30 (50.0%), respectively.

**TABLE 7:** Hypertension rate before and after overlap weighting.

Treatment group	Time point	Unweighted rate (n/N)	Unweighted 95% CI	Overlap-weighted rate	Weighted 95% CI
Roxadustat	Baseline	70.0% (21/30)	50.4–84.6	61.1%	41.1–81.0
Roxadustat	Month 3	76.7% (23/30)	57.3–89.4	82.6%	67.0–98.1
Roxadustat	Month 6	70.0% (21/30)	50.4–84.6	60.7%	40.7–80.7
Mircera	Baseline	53.3% (16/30)	34.6–71.2	61.1%	40.3–81.8
Mircera	Month 3	56.7% (17/30)	37.7–74.0	57.6%	36.5–78.6
Mircera	Month 6	50.0% (15/30)	33.2–66.8	57.5%	36.4–78.6

Unweighted rates are observed proportions and retain the original event count/available denominator. Overlap-weighted rates are ATO-weighted estimates and are presented without n/N. CI, confidence interval.

After overlap weighting, hypertension rates were 61.1% in both the roxadustat group and the Mircera group at baseline. At month 3, weighted hypertension rates were 82.6% in the roxadustat group and 57.6% in the Mircera group. At month 6, weighted hypertension rates were 60.7% and 57.5%, respectively.

In the weighted GEE model, the month 3 time effect in the roxadustat reference group had an RR of 1.35 (0.97–1.88) (P = 0.071). The month 3 treatment-by-time interaction RR was 0.70 (0.43–1.12) (P = 0.138), and the prespecified month 6 treatment-by-time interaction RR was 0.95 (0.57–1.57) (P = 0.834) (Table 8).

**TABLE 8:** Overlap-weighted GEE model for hypertension rate.

Effect	Weighted RR (95% CI)^a^	P Value
Group effect (Mircera vs. roxadustat)	1.00 (0.62–1.61)	1.000
Time effect (month 3 vs. baseline)	1.35 (0.97–1.88)	0.071
Time effect (month 6 vs. baseline)	0.99 (0.80–1.24)	0.956
Group x time (Mircera vs. roxadustat, month 3 vs. baseline)	0.70 (0.43–1.12)	0.138
Group x time (Mircera vs. roxadustat, month 6 vs. baseline)	0.95 (0.57–1.57)	0.834

^a^ The model was an overlap-weighted GEE, model including treatment group, time, and treatment-by-time interaction; covariates balanced through overlap weighting were not additionally adjusted.

The group effect represents the between-group difference at baseline; time effects represent changes from baseline in the reference group; group-by-time terms represent difference-in-differences. The month 6 interaction was the prespecified primary contrast. CI, confidence interval; GEE, generalized estimating equations; RR, risk ratio; exchangeable correlation structure.

### 3.6 Recorded complications

Recorded complications were sparse and are summarized descriptively in Table 9. Hypertension was recorded as a complication in 4 roxadustat-treated patients and 4 Mircera-treated patients. One hypertensive intracerebral hemorrhage event was recorded in the Mircera group. Arteriovenous fistula stenosis, arteriovenous fistula occlusion, and dialyzer clotting were infrequent.

**TABLE 9:** Recorded complications during follow-up.

Treatment group	Complication	n
Mircera	Not recorded	22
Mircera	Hypertension	4
Mircera	Arteriovenous fistula stenosis	2
Mircera	Arteriovenous fistula occlusion	1
Mircera	Hypertensive intracerebral hemorrhage	1
Roxadustat	Not recorded	23
Roxadustat	Hypertension	4
Roxadustat	Arteriovenous fistula occlusion	1
Roxadustat	Arteriovenous fistula stenosis	1
Roxadustat	Dialyzer clotting	1

Complications were summarized descriptively because the dataset contained sparse event records.

### 3.7 Sensitivity analyses

In the unweighted multivariable MMRM sensitivity analysis, the Hb model adjusted for baseline Hb, age, sex, baseline PTH, baseline CRP, dialysis vintage, prior ESA type/dose, baseline ferritin, and baseline TRF. The 6-month LS mean difference in Hb change for Mircera versus roxadustat was 8.64 (1.30, 15.98) (P = 0.022) (Supplementary Table S1). In the unweighted GEE sensitivity analyses, the binary models included treatment group, time, and treatment-by-time interaction. The month 6 treatment-by-time interaction RR was 1.63 (0.54–4.98) (P = 0.389) for Hb target attainment and 0.94 (0.61–1.43) (P = 0.765) for hypertension (Supplementary Tables S2, S3).

## 4 Discussion

In this single-center retrospective cohort of maintenance hemodialysis patients with renal anemia, overlap-weighted analysis showed a larger 6-month increase in Hb with Mircera than with roxadustat. The weighted MMRM estimated a 6-month LS mean difference in Hb change of 8.27 (1.87, 14.67) favoring Mircera. The unweighted multivariable Hb sensitivity analysis, which adjusted for baseline Hb, age, sex, baseline PTH, baseline CRP, dialysis vintage, prior ESA type/dose, baseline ferritin, and baseline TRF, produced a directionally consistent estimate (8.64 (1.30, 15.98); P = 0.022). In contrast, Hb target attainment defined strictly as 110–130 g/L and hypertension rates did not show statistically significant 6-month treatment-by-time interaction effects in the weighted GEE models. To our knowledge, this is the first head-to-head comparison study of CERA and roxadustat in Chinese maintenance hemodialysis patients.

The Hb finding should be interpreted in relation to, but not conflated with, the randomized roxadustat literature. Roxadustat increased or maintained Hb in Chinese dialysis and non-dialysis CKD phase 3 trials (Chen et al., 2019b; Chen et al., 2019a). ROCKIES and pooled dialysis-dependent analyses have also supported its efficacy in dialysis-dependent CKD (Fishbane et al., 2022; Barratt et al., 2021). Incident-dialysis analyses and non-dialysis pooled analyses extend the broader trial context (Provenzano et al., 2021a; Provenzano et al., 2021b; Provenzano et al., 2021c). Pooled safety and iron-parameter analyses further define the clinical profile of roxadustat (Barratt et al., 2023; Ganz et al., 2023), and thromboembolic-risk and inflammation-stratified analyses show that outcomes can vary with patient context and trial population definitions (Hamano et al., 2024; Chouk et al., 2025). The present study differs from those trials in several important ways: treatment was chosen in routine practice, the sample was small, prior ESA type/dose was unevenly distributed before weighting, and inflammatory and iron-related variables were incompletely captured. Therefore, the observed smaller roxadustat Hb response should be viewed primarily as a real-world comparative finding within an overlap-weighted target population, not as evidence that contradicts the randomized efficacy of roxadustat.

The Mircera result is biologically and clinically plausible in the context of prior long-acting ESA studies. Methoxy polyethylene glycol-epoetin beta has maintained Hb control in dialysis populations when administered every 2 weeks (Klinger et al., 2007; Levin et al., 2007), and randomized evidence has also addressed cardiovascular safety relative to other ESAs (Locatelli et al., 2019). Monthly or once-monthly maintenance strategies have also been evaluated in hemodialysis patients (Carrera et al., 2010; Duman et al., 2014). In a cohort containing many ESA-switching patients, a long-acting injectable ESA may also benefit from familiar dialysis-unit administration workflows and established dose-adjustment practices. However, the same clinical familiarity can generate selection bias: patients selected for Mircera may have differed from roxadustat-treated patients in ways that are not fully measured, including clinician expectations, prior ESA responsiveness, inflammation, iron availability, and adherence-related considerations.

Several measured factors were explicitly incorporated into the overlap-weighting design to address the reviewers’ concern about selection bias. The propensity-score model included baseline Hb, age, sex, primary kidney disease, hypertension at baseline, baseline PTH, body weight, baseline CRP, dialysis vintage, prior ESA type/dose, baseline ferritin, and baseline TRF. Baseline CRP was included because even modest inflammatory elevation has been associated with ESA hyporesponsiveness in hemodialysis patients (Kimachi et al., 2015). Prior ESA type/dose and dialysis vintage were included because anemia-treatment response and dose requirements can vary according to treatment history and dialysis context (Akizawa et al., 2021; Fujii et al., 2023; Hejaili et al., 2017). Baseline hypertension was included because blood pressure management is a persistent clinical issue in maintenance hemodialysis and may influence treatment selection and safety interpretation (Georgianos and Agarwal, 2021).

The primary weighted outcome model adjusted for treatment group, time, treatment-by-time interaction, and baseline Hb, while the other measured confounders were balanced through the weighting design rather than added again as model covariates. This approach is consistent with the design-analysis separation commonly recommended for propensity-score methods: first create a comparison with improved measured-covariate balance (Austin, 2011; Austin, 2009), then estimate treatment contrasts in the weighted overlap population (Li et al., 2019; Thomas et al., 2020). Because the analysis remained observational, however, overlap weighting cannot address unmeasured confounding. The persistent possibility of residual selection bias is especially important here because treatment allocation was partly driven by routine practice and patient preference rather than randomization.

The binary outcomes add nuance to the Hb result. Most maintenance hemodialysis (MHD) patients complicated with renal anemia in our center were initially treated with roxadustat, which is prone to cause substantial fluctuations in hemoglobin levels. Hb target attainment was defined as the Accordingly, target range of 110–130 g/L, consistent with the safety lessons from earlier ESA trials showing that higher Hb correction strategies require caution (Besarab et al., 1998; Singh et al., 2006; Pfeffer et al., 2009). But this target range differs from the KDIGO 2026 guideline recommendation against Hb levels exceeding 11.5 g/dL, which represents one limitation of our study. lThe CREATE trial provides additional evidence that higher Hb correction does not necessarily translate into improved clinical outcomes (Drüeke et al., 2006). Weighted descriptive rates increased more numerically in the Mircera group, but the month 6 weighted treatment-by-time interaction was not statistically significant. This is not surprising given the small sample size, the limited number of target events, and the fact that target attainment is a narrower endpoint than mean Hb change: a patient can have a clinically meaningful Hb increase and still remain below target, or exceed the upper bound and no longer count as target-achieving. Similarly, hypertension did not show a significant 6-month differential change, but confidence intervals were wide and the study was not powered for safety outcomes.

The roxadustat adherence analysis was added to address a specific interpretive concern. At the patient level, the median dispensed/prescribed dose ratio was high, and most roxadustat-treated patients had ratios at or above conventional adherence thresholds. The mean ratio and distribution of dose-adjustment counts were reported because the median alone was not sufficiently informative. These findings suggest that gross dispensing nonadherence is unlikely to be the sole explanation for the Hb difference, although dispensing-based adherence remains an imperfect proxy for actual ingestion. Follow-up iron indices and administered iron-dose data were not available in the final dataset, which limits the ability to evaluate whether iron availability contributed to differential Hb response. This limitation is relevant because iron handling is central to both ESA-based treatment and HIF-PHI therapy (Macdougall et al., 2019; Ganz et al., 2023).

This study has several limitations. First, the sample included only 60 patients from a single center, limiting precision and generalizability. Second, treatment allocation was nonrandom and reflected routine care; even with overlap weighting, residual confounding remains the main competing explanation for the observed Hb difference. Third, baseline transferrin saturation, follow-up TSAT, serum iron, longitudinal ferritin changes, and actual administered iron doses were unavailable, so the analysis could not fully characterize iron status during follow-up. Fourth, baseline covariate missingness was addressed by k-nearest-neighbor imputation before weighting; although this preserved sample size, it cannot restore information that was not recorded. Fifth, recorded complications were sparse and should be interpreted descriptively rather than as comparative safety evidence.

Despite these limitations, the study has practical strengths. It used the final patient-level dataset, incorporated reviewer-specified covariates into the overlap-weighting model, retained all 60 patients after k-nearest-neighbor imputation of baseline covariates, applied repeated-measures models to use both 3-month and 6-month Hb change data, and presented both unweighted and overlap-weighted descriptive estimates. The results are therefore best viewed as hypothesis-generating evidence from a carefully adjusted real-world comparison. They support the need for larger prospective studies or registry-based analyses with complete iron-treatment data, adjudicated safety outcomes, and prespecified handling of ESA-naive and ESA-switching subgroups.

## 5 Conclusion

In this single-center retrospective cohort, overlap-weighted analysis suggested a larger 6-month Hb increase with Mircera than with roxadustat. Hb target attainment and hypertension-rate analyses did not show statistically significant 6-month treatment-by-time interactions. These findings should be interpreted cautiously given small sample size, nonrandom treatment selection, and incomplete iron-related follow-up data.

## Data Availability

The raw data supporting the conclusions of this article will be made available by the authors, without undue reservation.
