# Supplementary material for: Comparative efficacy and safety of methoxy polyethylene glycol-epoetin beta and roxadustat in the management of renal anemia in maintenance hemodialysis
Source: Front Pharmacol. 2026 Aug 20;17:1862251. doi: 10.3389/fphar.2026.1862251 (PMC13537994; doi:10.3389/fphar.2026.1862251)
Supplement: Supplementary file 1 [file Table1.docx]

Supplementary Table 1. Unweighted multivariable MMRM sensitivity analysis for Hb change at 6 months

| **Characteristic** | **Roxadustat (N=30)** | **Mircera (N=30)** |
| --- | --- | --- |
| **Baseline Hb (g/L)** |  |  |
| n | 30 | 30 |
| Mean (SD) | 96.1 (14.82) | 96.6 (15.97) |
| **6-month Hb (g/L)** |  |  |
| n | 30 | 30 |
| Mean (SD) | 102.4 (14.80) | 112.1 (11.14) |
| **Change from baseline** |  |  |
| n | 30 | 30 |
| Mean (SD) | 6.3 (18.67) | 15.5 (16.56) |
| LS mean change (SE), a | 7.16 (2.829) | 15.81 (2.999) |
| **Between-group comparison** |  |  |
| Mircera - roxadustat |  |  |
| Difference in LS mean (95% CI), a | 8.64 (1.30, 15.98) |  |
| P value | 0.022 |  |
| *a The unweighted sensitivity model adjusted for baseline Hb, age, sex, baseline PTH, baseline CRP, dialysis vintage, prior ESA type/dose, baseline ferritin, and baseline TRF.* | | |
| *CI = confidence interval; Hb = hemoglobin; LS = least squares; MMRM = mixed model for repeated measures; SD = standard deviation.* | | |
