# Supplementary material for: Comparative efficacy and safety of methoxy polyethylene glycol-epoetin beta and roxadustat in the management of renal anemia in maintenance hemodialysis
Source: Front Pharmacol. 2026 Aug 20;17:1862251. doi: 10.3389/fphar.2026.1862251 (PMC13537994; doi:10.3389/fphar.2026.1862251)
Supplement: Supplementary file 2 [file Table2.docx]

Supplementary Table 2. Unweighted GEE sensitivity analysis for Hb target attainment (110-130 g/L)

| **Effect** | **Unweighted RR (95% CI), a** | **P value** |
| --- | --- | --- |
| Group effect (Mircera vs roxadustat) | 0.86 (0.33–2.25) | 0.754 |
| Time effect (month 3 vs baseline) | 1.00 (0.41–2.42) | 1.000 |
| Time effect (month 6 vs baseline) | 1.43 (0.66–3.11) | 0.368 |
| Group x time (Mircera vs roxadustat, month 3 vs baseline) | 1.83 (0.56–6.05) | 0.320 |
| **Group x time (Mircera vs roxadustat, month 6 vs baseline)** | **1.63 (0.54–4.98)** | **0.389** |
| *a The model was an unweighted GEE sensitivity analysis including treatment group, time, and treatment-by-time interaction.* | | |
| *The group effect represents the between-group difference at baseline; time effects represent changes from baseline in the reference group; group-by-time terms represent difference-in-differences. The month 6 interaction was the prespecified primary contrast. CI = confidence interval; GEE = generalized estimating equations; RR = risk ratio; exchangeable correlation structure.* | | |
