# Supplementary material for: Comparative efficacy and safety of methoxy polyethylene glycol-epoetin beta and roxadustat in the management of renal anemia in maintenance hemodialysis
Source: Front Pharmacol. 2026 Aug 20;17:1862251. doi: 10.3389/fphar.2026.1862251 (PMC13537994; doi:10.3389/fphar.2026.1862251)
Supplement: Supplementary file 3 [file Table3.docx]

Supplementary Table 3. Unweighted GEE sensitivity analysis for hypertension rate

| **Effect** | **Unweighted RR (95% CI), a** | **P value** |
| --- | --- | --- |
| Group effect (Mircera vs roxadustat) | 0.76 (0.51–1.15) | 0.192 |
| Time effect (month 3 vs baseline) | 1.10 (0.85–1.41) | 0.480 |
| Time effect (month 6 vs baseline) | 1.00 (0.83–1.21) | 1.000 |
| Group x time (Mircera vs roxadustat, month 3 vs baseline) | 0.97 (0.65–1.45) | 0.883 |
| **Group x time (Mircera vs roxadustat, month 6 vs baseline)** | **0.94 (0.61–1.43)** | **0.765** |
| *a The model was an unweighted GEE sensitivity analysis including treatment group, time, and treatment-by-time interaction.* | | |
| *The group effect represents the between-group difference at baseline; time effects represent changes from baseline in the reference group; group-by-time terms represent difference-in-differences. The month 6 interaction was the prespecified primary contrast. CI = confidence interval; GEE = generalized estimating equations; RR = risk ratio; exchangeable correlation structure.* | | |
